# Draft Genome Sequence of Saccharomyces cerevisiae LW2591Y, a Laboratory Strain for *In Vivo* Multigene Assemblies

**DOI:** 10.1128/MRA.01418-20

**Published:** 2021-03-04

**Authors:** Jennifer Gerke, Dominik Schneider, Anja Poehlein, Virginia W. Cornish, Gerhard H. Braus

**Affiliations:** a Molecular Microbiology and Genetics, Institute of Microbiology and Genetics, Georg-August-Universität Göttingen, Göttingen, Germany; b Genomic and Applied Microbiology & Göttingen Genomics Laboratory, Institute of Microbiology and Genetics, Georg-August-Universität Göttingen, Göttingen, Germany; c Department of Chemistry, Columbia University, New York, New York, USA; Vanderbilt University

## Abstract

Saccharomyces cerevisiae is an industrially preferred cell factory for the heterologous production of proteins and chemicals. Here, we present the draft genome sequence of the laboratory strain Saccharomyces cerevisiae LW2591Y, which has been designed for robust and efficient assembly of multigene pathways.

## ANNOUNCEMENT

Saccharomyces cerevisiae LW2591Y has been developed for the *in vivo* chromosomal assembly of multigene pathways for synthetic biology (1). The reiterative recombination assembly method is a simple, efficient, and robust way to assemble an indefinite number of DNA constructs. Recently, LW2591Y was used for the heterologous production of the fragrance geraniol (2) and for the development of a colorimetric assay to detect pathogen-derived peptides (3).

LW2591Y was cultivated in yeast-extract-peptone-dextrose medium overnight, and total genomic DNA was isolated by potassium acetate extraction (2). Illumina shotgun libraries were prepared using the NEBNext Ultra II DNA library prep kit with beads (New England Biolabs). Sequencing was performed on a MiSeq system with the v3 reagent kit (Illumina) with 600 cycles, resulting in 6,729,204 paired-end reads. For long-read sequencing, libraries were prepared from high-molecular-weight DNA using the ligation sequencing kit 1D (SQK-LSK109) and the native barcode expansion kit (barcode 7, EXP-NBD104; barcode 14, EXP-NBD114) (Oxford Nanopore Technologies). Sequencing was performed twice on a MinION Mk1B device and a SpotON R9.4.1 flow cell (Oxford Nanopore Technologies) for 96 h using MinKNOW v19.12.5 software for sequencing and Guppy v4.0.15 for demultiplexing and base calling, resulting in 786,167 reads.

Default parameters were used for all software unless otherwise specified. Short-reads were quality filtered by fastp v0.20.0 (4) (base Phred score, ≥Q20; correction by overlap, read clipping with a sliding window of 4 with a Phred score of Q ≥ 20; required minimum length, ≥50 bp). Reads were adapter trimmed by Cutadapt v2.5 (5). Potential phiX contamination was removed by mapping the quality-filtered short reads against another sequence (GenBank accession number NC_001422) using Bowtie 2 v2.3.5.1 (6). The Nanopore reads were processed with fastp v0.20.0 (4) (base Phred score, ≥Q10; clipping by sliding window of 10 with a Phred score of ≥Q10; required minimum length, ≥5,000 bp), followed by Porechop v0.2.4 (https://github.com/rrwick/Porechop). After processing, 6,525,536 short reads with an average length of 257 bp and 387,790 long reads with an average length of 9,314 bp (*N*_50_, 9,753 bp [7]) were obtained.

*De novo* hybrid assembly was performed with Unicycler v0.4.8 (8) in normal mode. Contigs with less than 200 bp were removed. The assembly resulted in 29 contigs, which were aligned using BLASTn (9) with the Saccharomyces cerevisiae S288C reference chromosomes and mitochondrial DNA (GenBank accession number GCA_000146045.2), as well as a 2-μm plasmid (J01347.1). The 27 contigs assigned to the S288C chromosomes were subsequently mapped with Mauve v20150226 (build 10) (10) and Lasergene v17.1 (DNASTAR, Madison, WI) and scaffolded manually by adding 100 Ns. Short contigs mapping to repetitive elements of S288C chromosome 12 were inserted only once.

The draft genome sequence comprises 11,853,285 bp with an *N*_50_ value of 799,432 bp (7). Coverages were estimated with QualiMap v2.2.2 (11) using Bowtie 2 v2.3.5.1 (6) and minimap2 v2.17-r941 (12). The short-read coverages were 113× for the chromosomal DNA, 2,654× for the mitochondrial DNA, and 424× for the plasmid DNA. The long-read coverages were 278× for the chromosomal DNA, 2,148× for the mitochondrial DNA, and 259× for the plasmid DNA. The total GC content is 38.1%.

A breseq v0.35.0 analysis (2, 13) of the short reads against the reference strain S288C revealed 235 single nucleotide polymorphisms and insertions/deletions with 100% frequency (see Table S1 at https://figshare.com/s/acf5207e3169bc9e8fc1). In 56 genes, the mutations induce changes in the corresponding amino acid sequences.

### Data availability.

The data for Saccharomyces cerevisiae LW2591Y have been deposited at GenBank under accession numbers CP059522 through CP059539, with BioProject accession number PRJNA611915 and SRA accession numbers SRX9268844 (Oxford Nanopore) and SRX7891670 (MiSeq).
